# Hedonic Hunger and Problematic Internet Use Show Distinct Associations with General and Central Adiposity in Medical Students

**DOI:** 10.3390/healthcare14162483

**Published:** 2026-08-11

**Authors:** Ozge Kama Basci, Hasan Huseyin Zorlu

**Affiliations:** 1 Department of Internal Medicine, Faculty of Medicine, Balikesir University, Altieylul, Balikesir 10130, Türkiye; 2 Department of Internal Medicine, Genç State Hospital, Bingol 12500, Türkiye; zorluhasanhuseyin@gmail.com

**Keywords:** hedonic hunger, problematic internet use, medical students, obesity, Power of Food Scale, waist circumference

## Abstract

**Background:** Hedonic hunger and problematic internet use (PIU) have been linked to excess adiposity, but their differential associations with adiposity measures in medical students remain unclear. **Methods:** This cross-sectional study included 256 medical students (63.3% female; mean age 22.1 ± 2.4 years) at Balıkesir University in 2024. Hedonic hunger was assessed using the Power of Food Scale (PFS), and PIU using the Problematic Internet Use Scale (PIUS). Anthropometric measurements included body mass index (*BMI*), waist circumference, and waist-to-hip ratio (WHR). Multivariable linear and binary logistic regression analyses were performed, together with secondary correlation and group-comparison analyses. **Results:** Overall, 32.1% of participants had overweight or obesity. Mean PFS and PIUS total scores were 3.25 ± 0.73 and 2.61 ± 0.74, respectively. In multivariable models, higher PFS scores were associated with greater *BMI* (*B* = 1.32, *p* = 0.001) and higher odds of overweight/obesity (*OR* = 1.79, 95% CI: 1.13–2.84). Higher PIUS scores were associated with waist circumference (*B* = 3.85, *p* < 0.001) and WHR (*B* = 0.023, *p* = 0.027), but not with *BMI*. The association between PIUS and waist circumference was stronger in male students (PIUS × sex interaction, *p* = 0.006). PFS and PIUS scores were positively correlated (*r* = 0.299, *p* < 0.001). **Conclusions:** Hedonic hunger and PIU showed different cross-sectional associations with adiposity measures: hedonic hunger was associated primarily with *BMI* and overweight/obesity status, whereas PIU was associated with central adiposity measures. The association between PIU and waist circumference differed by sex. Longitudinal studies are needed to clarify the temporal relationships among these variables.

## 1. Introduction

Obesity is one of the most pressing public health challenges of the 21st century. The World Obesity Atlas 2023 predicts that approximately 24% of the global population—around two billion individuals—will be living with obesity by 2035 [1]. Global trends show a particularly steep increase among young adults and students. A 2024 systematic review and meta-analysis by Shafiee et al. encompassing 47,455 medical students from 99 studies worldwide reported a pooled overweight prevalence of 18% (95% CI: 17–20%) and obesity prevalence of 9% (95% CI: 7–11%), with meta-regression demonstrating a significant upward trend over time [2]. In Turkey, the combined prevalence of overweight and obesity in young adults aged 15–24 years was reported to be 19.5% in the 2019 Turkey Nutrition and Health Survey [3].

The pathophysiology of obesity is complex. Alongside classical contributors—physical inactivity, caloric surplus, and metabolic factors—behavioral drivers of food intake have received increasing research attention. Hedonic hunger, a concept formalized by Lowe and Butryn, refers to the motivational drive to consume palatable, energy-dense food in the absence of homeostatic energy need, driven by reward and pleasure-seeking rather than physiological deficit [4]. The Power of Food Scale (PFS), developed to operationalize hedonic hunger, assesses an individual’s psychological responsiveness to a food-abundant environment across three levels of proximity to food: food available in the environment but not physically present (FA), food present but not yet tasted (FP), and food tasted but not fully consumed (FT) [5]. Karamizadeh et al.’s 2024 systematic review and meta-analysis of 25 studies (N = 14,457 adults) confirmed a positive, albeit weak, association between PFS scores and *BMI* [6].

Problematic internet use (PIU) has emerged as a significant behavioral health concern, particularly among university students. It is characterized by loss of control over internet use with consequent impairment in daily functioning, social relationships, and psychological well-being [7]. University students are at elevated risk due to unrestricted internet access, academic pressure, and social connectivity demands [8]. A systematic review and meta-analysis by Aghasi et al. comprising 38,537 participants demonstrated that high internet use was associated with 47% greater odds of overweight or obesity (OR: 1.47; 95% CI: 1.21–1.78), with each additional hour per day of internet use carrying an 8% increased odds [9]. More recently, a 2025 study of 610 medical students reported that internet addiction statistically mediated the association between overweight/obesity and psychological distress, highlighting potential interrelations among PIU, weight status, and mental health in this specific population [10].

Although previous studies have linked problematic internet use with disordered eating behaviors, evidence specifically addressing hedonic hunger, as measured by the Power of Food Scale, in relation to problematic internet use among medical students remains limited. Medical students represent a population exposed to unique academic and psychosocial demands, making the coexistence of these behavioral characteristics particularly relevant to investigation. In addition, evidence examining hedonic hunger and problematic internet use simultaneously in relation to both general adiposity (*BMI*) and central adiposity (waist circumference and waist-to-hip ratio) within the same cohort remains limited. This distinction is important because general and central adiposity reflect different aspects of body composition and differ in their associations with cardiometabolic risk [11]. Examining these behavioral constructs across multiple anthropometric measures may provide a more comprehensive understanding of their relationships with adiposity. Accordingly, the present study aimed to evaluate hedonic hunger and problematic internet use simultaneously in medical students and to examine their associations with general and central adiposity, together with relevant anthropometric indices and lifestyle characteristics.

## 2. Materials and Methods

### 2.1. Study Design and Ethics

This cross-sectional analytical study was conducted at Balıkesir University Faculty of Medicine (BAUN) in Turkey, in 2024. Ethical approval was granted by the BAUN Non-Invasive Research Ethics Committee (Decision No: 2024/11; Date: 31 January 2024). The study adhered to the principles of the Declaration of Helsinki. Written informed consent was obtained from all participants prior to data collection.

### 2.2. Participants and Sampling

The target population comprised all students enrolled across the six academic years of the BAUN Faculty of Medicine in 2024, corresponding to 1010 eligible students. The survey instrument was administered electronically via Google Forms and disseminated to students through class-year representatives; over a three-month period, a survey announcement was made once per month to each class-year while students were together in their classrooms between lectures. Participation was voluntary, and students who completed the online survey were subsequently invited to the Internal Medicine outpatient clinic for anthropometric measurements. A total of 256 students completed the survey and underwent anthropometric assessment, corresponding to a participation rate of 25.3% (256/1010). Because demographic and academic data were not systematically collected for non-participants, a formal comparison between participants and non-participants could not be performed; this is acknowledged as a limitation. An a priori power analysis was conducted using G*Power 3.1 for the primary multivariable linear regression models, the main analyses of this study, each incorporating 8 predictors. Using a linear multiple regression (fixed model, R^2^ deviation from zero) test with a medium effect size (*f*^2^ = 0.15), α = 0.05, and power = 0.80, the minimum required sample size was 109. The achieved sample of 256 therefore exceeded this requirement, providing adequate power to detect the hypothesized associations. This power analysis was based on the three primary multivariable linear regression models (Models 1–3); Model 4, the complementary logistic regression analysis of overweight/obesity status, was not the basis for this a priori power calculation. Students with incomplete questionnaire data or without complete anthropometric assessment were excluded. In addition to the PFS and PIUS instruments, the survey collected self-reported sociodemographic and lifestyle information, including age, sex, curricular year, current smoking status, alcohol use, and regular exercise. Regular exercise was assessed with a single yes/no item: “Do you exercise regularly (≥150 min/week of moderate-intensity exercise, or ≥75 min/week of vigorous-intensity exercise)?” No validated physical activity instrument (e.g., the International Physical Activity Questionnaire, Short Form) was used, and the frequency, duration, and type of exercise were not further quantified.

### 2.3. Power of Food Scale (PFS)

Hedonic hunger was assessed using the Turkish-validated 15-item PFS [5,12]. The scale measures psychological responsiveness to food-abundant environments across three subscales: FA (items 1,2,5,10,11,13—6 items), FP (items 3,4,6,7—4 items), and FT (items 8,9,12,14,15—5 items). Each item is scored on a 5-point Likert scale ranging from 1 (do not agree at all) to 5 (strongly agree). The PFS total score was the mean of the three subscale means. A total score of ≥2.5 is indicative of a higher level of hedonic hunger [5]. This threshold, adopted from the PFS literature, was used descriptively to facilitate comparison with previous studies employing the same convention; the PFS is a dimensional, non-diagnostic self-report measure, and this cut-off does not represent a clinical or diagnostic criterion. The Turkish adaptation has demonstrated satisfactory reliability and validity in university-based samples [12]. In the present sample, internal consistency was excellent for the PFS total score (Cronbach’s α = 0.899) and good to acceptable for the FA, FP, and FT subscales (α = 0.842, 0.755, and 0.804, respectively).

### 2.4. Problematic Internet Use Scale (PIUS)

PIU was evaluated using the PIUS (Turkish: PİKÖ), a 33-item instrument developed by Shapira et al. and validated in Turkish by Ceyhan et al. [13]. The scale encompasses three subscales: Negative Consequences of Internet Use (NC—17 items), Social Benefit/Social Comfort (SB—10 items), and Excessive Use (EU—6 items). Items are scored on a 5-point scale (1 = never to 5 = completely applicable). Two items were reverse-scored. Higher total scores indicate greater problematic use. The PIUS is not a diagnostic tool but quantifies the severity of problematic use as a continuous measure. In the present sample, internal consistency was excellent for the PIUS total score (Cronbach’s α = 0.955) and good to excellent for the EU, SB, and NC subscales (α = 0.809, 0.873, and 0.946, respectively).

### 2.5. Anthropometric Measurements

Height (cm) and weight (kg) were measured using a calibrated stadiometer/scale (SECA 284, SECA GmbH & Co. KG, Hamburg, Germany) by an internal medicine physician. Waist circumference (cm, at the midpoint between the inferior costal margin and the iliac crest) and hip circumference (cm, at the level of the greater trochanters) were measured according to WHO standard recommendations. The first 10 participants were measured jointly by two observers as a procedural standardization exercise; however, no formal inter-rater reliability coefficient was calculated, and all remaining measurements were performed by a single trained internal medicine physician. *BMI* was calculated as weight (kg) divided by height squared (m^2^) and categorized according to WHO criteria: underweight (<18.5), normal weight (18.5–24.9), overweight (25.0–29.9), class I obese (30.0–34.9), class II obese (35.0–39.9), and class III obese (≥ 40.0 kg/m^2^).

### 2.6. Statistical Analysis

Data were analyzed using IBM SPSS Statistics 24.0 (IBM Corp., Armonk, NY, USA). Continuous variables are presented as mean ± standard deviation (SD), and categorical variables are presented as frequencies and percentages. Independent-samples *t*-tests were used to compare two independent groups, and one-way analysis of variance (ANOVA) was used to compare three or more independent groups. For variables showing a significant overall difference, post hoc tests were applied for pairwise between-group comparisons, using Bonferroni correction. Categorical associations were examined with the chi-square test. Effect sizes were reported alongside *p*-values: partial eta-squared (η^2^*p*) for ANOVA/Welch’s ANOVA comparisons (Table 1 and Supplementary Tables S1 and S4) and Cohen’s d for independent-samples *t*-test comparisons (Table 3 and Supplementary Table S2). The online questionnaire required a response to every item, and forms with incomplete responses were excluded prior to analysis (see Participants and Sampling); the analytic dataset therefore contained no missing data, and no imputation procedure was required. Bivariate correlations were evaluated with Pearson’s correlation coefficient. Internal consistency of the PFS and PIUS total scores and subscales was assessed using Cronbach’s alpha. For the multivariable linear and logistic regression models, multicollinearity among predictors was evaluated using variance inflation factors (VIFs) and tolerance values. Residuals were also examined using the Breusch–Pagan test for heteroscedasticity. Because heteroscedasticity was detected for the waist circumference model, a sensitivity analysis using heteroscedasticity-consistent (HC3) robust standard errors was performed for this model. Because waist circumference and waist-to-hip ratio are strongly influenced by sex and body size, a PIUS × sex interaction term was additionally entered into these two models, and sex-stratified regression analyses were performed to further characterize the association between problematic internet use and central adiposity. As a further sensitivity analysis, the three primary linear regression models (Models 1–3) were re-estimated using heteroscedasticity-consistent (HC3) robust standard errors, and the complementary logistic regression model (Model 4) was re-estimated using robust (sandwich-type) standard errors; separately, non-parametric bootstrap resampling (1000 resamples) was used to obtain bootstrap 95% confidence intervals for the primary exposure coefficients (PFS total and PIUS total) across all four models. None of the regression models included measures of dietary intake, physical activity beyond the binary exercise variable, sleep duration or quality, or psychological stress/anxiety, as these were not collected; this represents a major limitation of the applied models, as residual confounding by these factors cannot be excluded and the reported coefficients should be interpreted accordingly (see Limitations). Statistical significance was set at α = 0.05. Predictors for the multivariable regression models were selected a priori on theoretical and empirical grounds rather than by statistical selection procedures: PFS total and PIUS total scores were entered as the primary exposures of interest, while sex, age, curricular stage (preclinical/clinical), smoking status, alcohol use, and exercise were included as covariates on the basis of their established associations with adiposity and with hedonic eating and internet-use behaviors in the existing literature. All eight predictors were entered simultaneously (forced entry) in each model; no stepwise, backward, or other data-driven selection procedure was used, so as to avoid selection bias and to allow interpretation of each predictor’s association independent of the others. Normality of continuous variables was assessed using the Shapiro–Wilk test, and homogeneity of variance for all independent-samples *t*-test and ANOVA comparisons (Tables 1 and 3, and Supplementary Tables S1 and S4) was assessed using Levene’s test. Although Shapiro–Wilk testing indicated departures from normality for most continuous variables, parametric tests were retained given the overall sample size (*N* = 256) and the Central Limit Theorem; where Levene’s test indicated unequal variances (*p* < 0.05), Welch’s *t*-test was used in place of Student’s *t*-test, and Welch’s ANOVA with Games–Howell post hoc comparisons was used in place of the classical ANOVA with Bonferroni-corrected post hoc comparisons. As a sensitivity analysis, statistically significant Pearson’s correlations were additionally checked against Spearman’s rank-order correlations; correlations not confirmed by both methods are noted accordingly in Table 2 and Supplementary Table S3. The multivariable linear regression models (Models 1–3, Table 4) were designated as the primary analyses of this study, and the multivariable logistic regression model (Model 4) was conducted as a complementary analysis of overweight/obesity status; the bivariate correlations (Table 2) and the additional subgroup and group comparisons (Tables 1 and 3) were treated as secondary, exploratory analyses conducted to characterize the dataset. Correction for multiple comparisons (Bonferroni) was applied only to the post hoc pairwise comparisons following a significant omnibus ANOVA; no correction for multiplicity was applied to the correlation coefficients or the additional group comparisons, given the large number of tests performed. These exploratory findings should therefore be interpreted as hypothesis-generating rather than confirmatory, and are discussed accordingly.

## 3. Results

A total of 256 medical students were included in this analysis. Of the participants, 162 (63.3%) were female and 94 (36.7%) were male, with a mean age of 22.11 ± 2.41 years (range: 18–38 years). According to curricular stage, 143 (55.9%) students were in the preclinical stage and 113 (44.1%) were in the clinical stage. Current smoking, alcohol consumption, and regular exercise were reported by 28.1%, 34.0%, and 39.8% of participants, respectively.

The mean *BMI* of the participants was 23.72 ± 4.12 kg/m^2^, and 82 (32.1%) students were overweight or obese (*BMI* ≥ 25 kg/m^2^). The mean waist circumference was 79.0 cm and mean hip circumference was 94.1 cm. Clinical-stage students had significantly higher *BMI* values than preclinical students (24.55 ± 4.06 vs. 23.07 ± 5.17 kg/m^2^, *p* = 0.013), and a greater proportion had *BMI* ≥ 25 kg/m^2^ (42.5% vs. 23.8%, *p* = 0.002). The academic year and curricular stage comparisons are presented in Supplementary Tables S1 and S2.

The mean PFS total score was 3.25 ± 0.73. Mean food available, food present, and food tasted scores were 2.87 ± 0.91, 3.36 ± 0.88, and 3.52 ± 0.78, respectively. The mean PIUS total score was 2.61 ± 0.74, whereas the mean excessive use, perceived social benefit, and negative consequences scores were 3.42 ± 0.88, 2.19 ± 0.80, and 2.20 ± 0.90, respectively.

The PFS and PIUS scores according to the *BMI* categories are presented in Table 1. Significant differences were observed across *BMI* categories for PFS total score (*p* = 0.001), food available (*p* < 0.001), food present (*p* < 0.001), PIUS total score (*p* = 0.009, Welch’s ANOVA), perceived social benefit (*p* < 0.001, Welch’s ANOVA), and negative consequences (*p* = 0.013, Welch’s ANOVA, though no individual pairwise comparison remained significant after Games–Howell correction). The omnibus difference in food tasted scores did not remain significant once Welch’s ANOVA was applied to account for unequal variances (*p* = 0.124), and is therefore not interpreted as a robust finding. Excessive use scores did not differ significantly among the *BMI* categories.

**Table 1:** Power of Food Scale and Problematic Internet Use Scale scores across *BMI* categories.

Variable	Underweight *BMI* < 18.5 (*n* = 16)	Normal Weight *BMI* 18.5–24.9 (*n* = 158)	Overweight *BMI* 25–29.9 (*n* = 56)	Obese *BMI* ≥ 30 (*n* = 26)	Total (*n* = 256)	*F*	*p*	Post Hoc
**PFS total**	**2.79 ± 0.84**	**3.21 ± 0.68**	**3.30 ± 0.75**	**3.67 ± 0.73**	**3.25 ± 0.73**	**5.45 (** $\eta_{p}^{2}$ ** = 0.061)**	**0.001**	**G1, G2 < G4 ^a^**
**PFS-FA**	**2.39 ± 0.96**	**2.77 ± 0.86**	**3.03 ± 0.91**	**3.44 ± 0.82**	**2.87 ± 0.91**	**6.57 (** $\eta_{p}^{2}$ ** = 0.073)**	**<0.001**	**G1, G2 < G4 ^a^**
**PFS-FP**	**2.98 ± 0.91**	**3.28 ± 0.85**	**3.40 ± 0.81**	**3.96 ± 0.90**	**3.36 ± 0.88**	**5.70 (** $\eta_{p}^{2}$ ** = 0.064)**	**<0.001**	**G1, G2, G3 < G4 ^a^**
PFS-FT	2.99 ± 0.95	3.58 ± 0.70	3.47 ± 0.94	3.61 ± 0.65	3.52 ± 0.78	2.02 ($\eta_{p}^{2}$ = 0.035)	0.124	ns (Welch)
**PIUS total**	**3.03 ± 0.59**	**2.52 ± 0.66**	**2.59 ± 0.79**	**2.93 ± 0.96**	**2.61 ± 0.74**	**4.37 (** $\eta_{p}^{2}$ ** = 0.050)**	**0.009**	**G2 < G1 ^b^**
PIUS-EU	3.65 ± 0.65	3.43 ± 0.80	3.33 ± 1.08	3.42 ± 0.99	3.42 ± 0.88	0.54 ($\eta_{p}^{2}$ = 0.006)	0.657	ns
**PIUS-SB**	**2.83 ± 0.80**	**2.04 ± 0.73**	**2.24 ± 0.74**	**2.63 ± 0.93**	**2.19 ± 0.80**	**7.04 (** $\eta_{p}^{2}$ ** = 0.094)**	**<0.001**	**G2 < G1 ^b^; G2 < G4 ^b^**
**PIUS-NC**	**2.62 ± 0.83**	**2.07 ± 0.81**	**2.18 ± 0.88**	**2.74 ± 1.20**	**2.20 ± 0.90**	**4.03 (** $\eta_{p}^{2}$ ** = 0.063)**	**0.013**	**ns (Games-Howell) ^c^**

**Note.** Values are presented as mean ± SD. One-way ANOVA with Bonferroni-corrected pairwise post hoc comparisons. G1 = Underweight (*BMI* < 18.5 kg/m^2^); G2 = Normal weight (*BMI* 18.5–24.9 kg/m^2^); G3 = Overweight (*BMI* 25–29.9 kg/m^2^); G4 = Obese (*BMI* ≥ 30 kg/m^2^). PFS = Power of Food Scale; PIUS = Problematic Internet Use Scale; FA = Food Available; FP = Food Present; FT = Food Tasted; EU = Excessive Use; SB = Social Benefit/Social Comfort; NC = Negative Consequences. Bold rows indicate significant omnibus F (*p* < 0.05). ^a^ Bonferroni-corrected *p* < 0.05. ns = not significant. Given departures from normality (Shapiro–Wilk *p* < 0.05) and, for several outcomes, unequal variances across *BMI* groups (Levene *p* < 0.05), Welch’s ANOVA with Games–Howell post hoc comparisons was used instead of the classical ANOVA/Bonferroni approach for PIUS total, PIUS-SB, and PIUS-NC, and for PFS-FT, whose omnibus difference did not remain significant under Welch’s ANOVA. ^b^ Games–Howell-corrected *p* < 0.05. ^c^ Omnibus Welch ANOVA significant, but no pairwise comparison remained significant after Games–Howell correction; interpret with caution. $\eta_{p}^{2}$ = partial eta-squared (effect size for the omnibus ANOVA/Welch’s ANOVA).

When participants were regrouped as normal/underweight, overweight, and obese, waist circumference, hip circumference, waist-to-hip ratio, PFS total score, food available, food present, and perceived social benefit scores differed significantly among groups (Supplementary Table S4). The negative consequences score also differed significantly under the classical ANOVA (*p* = 0.004), but this difference did not remain significant once Welch’s ANOVA was applied to account for unequal variances across groups (*p* = 0.052), and is therefore not interpreted as a robust finding. The excessive use scores did not differ significantly.

*BMI* was positively correlated with the PFS total score (*r* = 0.194, *p* = 0.002), food available (*r* = 0.254, *p* < 0.001), and food present (*r* = 0.221, *p* < 0.001). *BMI* was not correlated with the PIUS total score (*r* = 0.058, *p* = 0.377). Waist circumference was positively correlated with the PIUS total score (*r* = 0.294, *p* < 0.001), excessive use (*r* = 0.147, *p* = 0.019), perceived social benefit (*r* = 0.291, *p* < 0.001), and negative consequences (*r* = 0.324, *p* < 0.001). The detailed correlations are presented in Table 2.

**Table 2:** Correlations Between Anthropometric Indices, PFS, and PIUS Scores.

Variable 1	Variable 2	*r*	*p*
BMI	PFS total	0.194	0.002
	PFS-FA	0.254	<0.001
	PFS-FP	0.221	<0.001
	PIUS total	0.058	0.377
Waist circumference	Hip circumference	0.534	<0.001
	PFS total	0.130	0.038 ^d^
	PFS-FA	0.184	0.003
	PFS-FP	0.158	0.012 ^d^
	PIUS total	0.294	<0.001
	PIUS-EU	0.147	0.019
	PIUS-SB	0.291	<0.001
	PIUS-NC	0.324	<0.001
Hip circumference	PFS total	0.173	0.006
	PFS-FP	0.168	0.007
	PIUS total	0.124	0.048
	PIUS-NC	0.141	0.024
PFS total	PIUS total	0.299	<0.001
	PIUS-EU	0.290	<0.001
	PIUS-SB	0.199	0.001
	PIUS-NC	0.278	<0.001
PFS-FA	PIUS total	0.260	<0.001
	PIUS-EU	0.183	0.003
	PIUS-SB	0.205	0.001
	PIUS-NC	0.282	<0.001
PFS-FP	PIUS total	0.350	<0.001
	PIUS-EU	0.349	<0.001
	PIUS-SB	0.229	<0.001
	PIUS-NC	0.320	<0.001
PFS-FT	PIUS-EU	0.208	0.001

Note. BMI = body mass index; PFS = Power of Food Scale; PIUS = Problematic Internet Use Scale; FA = Food Available; FP = Food Present; FT = Food Tasted; EU = Excessive Use; SB = Social Benefit/Social Comfort; NC = Negative Consequences. Pearson’s correlation coefficients (*r*) are presented. Only statistically significant correlations are shown, except for the *BMI*–PIUS total correlation, which is reported because it was one of the main analyses. ^d^ Not confirmed by Spearman’s rank-order correlation (waist circumference–PFS total: ρ = 0.113, *p* = 0.072; waist circumference–PFS-FP: ρ = 0.116, *p* = 0.063); these Pearson correlations should be interpreted with caution.

Comparisons of PFS and PIUS scores according to sex, smoking, alcohol use, and exercise status are presented in Table 3. PFS scores did not differ by sex; however, male students had significantly higher PIUS total and Negative Consequences scores than females (*p* = 0.023 and *p* = 0.002, respectively). Because Levene’s test indicated unequal variances for several of these comparisons, Welch’s *t*-test was additionally applied as a sensitivity analysis: the sex differences in PIUS total and PIUS-NC remained significant (Welch’s *p* = 0.029 and *p* = 0.004, respectively), and male students also had significantly higher PIUS-SB (Social Benefit/Social Comfort) scores than females, a difference not identified as significant in the original equal-variance analysis (Welch’s *p* = 0.026). Smokers had significantly higher PFS-FP and PIUS-NC scores than non-smokers (Welch’s *p* = 0.035 and *p* = 0.021, respectively); no other significant differences were observed between smokers and non-smokers. Alcohol users had significantly higher PFS-FP scores than non-users (Welch’s *p* = 0.027); the PFS total difference was significant once Welch’s correction was applied (*p* = 0.0498), and the PFS-FA difference did not reach significance under Welch’s correction (*p* = 0.051), with no significant differences in PIUS scores. Students who exercised regularly had lower PFS total, PFS-FA, PFS-FP, PIUS total, PIUS-EU, and PIUS-NC scores than those who did not exercise regularly (Table 3).

**Table 3:** PFS and PIUS scores according to sex, smoking, alcohol use, and exercise status.

Variable	Group	*n* (%)	PFS Total	PFS-FA	PFS-FP	PFS-FT	PIUS Total	PIUS-EU	PIUS-SB	PIUS-NC
**Sex**	Female	162 (63.3)	3.23	2.85	3.34	3.51	2.52	3.41	2.10	2.07
	Male	94 (36.7)	3.28	2.91	3.39	3.55	2.74	3.46	2.35	2.42
	*p* value		NS (*d* = −0.07)	NS (*d* = −0.06)	NS (*d* = −0.06)	NS (*d* = −0.06)	**0.029 ^e^** (*d* = −0.30)	NS (*d* = −0.06)	0.026 ^e^ (*d* = −0.31)	**0.004 ^e^** (*d* = −0.40)
**Smoking**	Yes	72 (28.1)	3.33	2.90	3.56	3.61	2.75	3.54	2.28	2.42
	No	184 (71.9)	3.21	2.86	3.28	3.49	2.55	3.37	2.16	2.11
	*p* value		NS (*d* = 0.20)	NS (*d* = 0.04)	0.035 ^e^ (*d* = 0.31)	NS (*d* = 0.15)	NS (*d* = 0.27)	NS (*d* = 0.18)	NS (*d* = 0.14)	0.021 ^e^ (*d* = 0.35)
**Alcohol use**	Yes	87 (34.0)	3.39	3.04	3.54	3.59	2.68	3.49	2.27	2.28
	No	169 (66.0)	3.18	2.79	3.27	3.49	2.57	3.39	2.15	2.15
	*p* value		**0.0498 ^e^** (*d* = 0.28)	**NS (0.051) ^e^** (*d* = 0.28)	**0.027 ^e^** (*d* = 0.31)	NS (*d* = 0.12)	NS (*d* = 0.15)	NS (*d* = 0.11)	NS (*d* = 0.15)	NS (*d* = 0.15)
**Exercise**	Yes	102 (39.8)	3.09	2.74	3.13	3.41	2.42	3.17	2.08	2.01
	No	154 (60.2)	3.36	2.97	3.51	3.60	2.73	3.59	2.27	2.37
	*p* value		**0.005** (*d* = −0.36)	**0.047** (*d* = −0.26)	**0.001** (*d* = −0.44)	NS (d = −0.24)	**0.001** (*d* = −0.43)	**<0.001** (*d* = −0.49)	NS (*d* = −0.24)	**0.006** (*d* = −0.35)

**Note.** Values are presented as mean scores. PFS = Power of Food Scale; PIUS = Problematic Internet Use Scale; FA = Food Available; FP = Food Present; FT = Food Tasted; EU = Excessive Use; SB = Social Benefit/Social Comfort; NC = Negative Consequences; NS = not significant. Bold *p* values indicate statistical significance (*p* < 0.05). *p* values refer to independent-samples *t*-test comparisons between the two groups within each variable. ^e^ Levene’s test indicated unequal variances for this comparison; Welch’s (unequal-variances) *t*-test was used and is reported here in place of the original Student’s *t*-test result. *d* = Cohen’s *d* (effect size for the group difference; small ≈ 0.2, medium ≈ 0.5, large ≈ 0.8).

The PFS total score was positively correlated with the PIUS total score (*r* = 0.299, *p* < 0.001). Food availability (*r* = 0.260, *p* < 0.001) and food present (*r* = 0.350, *p* < 0.001) were correlated with the PIUS total score. Food tasted was correlated only with excessive use (*r* = 0.208, *p* = 0.001).

The results of the multivariate regression analyses are presented in Table 4. Higher PFS total scores were associated with higher *BMI* values (*B* = 1.321, β = 0.204, *p* = 0.001) and higher odds of overweight/obesity (*OR* = 1.788, 95% CI: 1.126–2.839, *p* = 0.014). PIUS total scores were associated with waist circumference (*B* = 3.853, β = 0.184, *p* < 0.001) and waist-to-hip ratio (*B* = 0.023, β = 0.123, *p* = 0.027). Female sex was associated with lower *BMI*, waist circumference, waist-to-hip ratio, and lower odds of overweight/obesity (all *p* < 0.001). Alcohol consumption was associated with *BMI*, waist circumference, and waist-to-hip ratio, whereas regular exercise was associated with lower odds of being overweight/obese (*OR* = 0.467, 95% CI: 0.236–0.927, *p* = 0.030). Multicollinearity diagnostics indicated no problematic collinearity among the predictors entered in Models 1–4: variance inflation factors ranged from 1.10 to 1.58 (tolerance values 0.63–0.91), well below the conventional threshold of concern (VIF > 5). Residual diagnostics for the linear regression models (Models 1–3) showed homoscedastic residuals for the *BMI* and waist-to-hip ratio models (Breusch–Pagan *p* = 0.599 and *p* = 0.449, respectively) but some evidence of heteroscedasticity for the waist circumference model (*p* = 0.005). Residuals for all three models showed modest departures from normality (Shapiro–Wilk *p* < 0.001), which is not unexpected given the sample size and is not expected to materially bias the coefficient estimates, though it warrants caution in interpreting the precision of the waist circumference model’s standard errors. To address this directly, a sensitivity analysis was conducted using heteroscedasticity-consistent (HC3) robust standard errors for the waist circumference model; this yielded materially unchanged estimates and inference, with the PIUS total coefficient remaining significant (*B* = 3.853, robust *SE* = 1.106, *p* <.001, robust 95% CI: 1.686–6.019) and closely matching the original ordinary-least-squares estimate (*SE* = 1.116, 95% CI: 1.655–6.051). In addition, because waist circumference and waist-to-hip ratio are strongly influenced by sex and body size, a PIUS × sex interaction term was added to the waist circumference and waist-to-hip ratio models, and sex-stratified regression analyses were performed. A significant PIUS × sex interaction was observed for waist circumference (*B* = −5.451, robust *SE* = 1.997, *p* = 0.006): the association between PIUS and waist circumference was significant within both sexes but was more pronounced among male students (*B* = 6.250, *SE* = 1.774, *p* < 0.001, 95% CI: 2.774–9.727) than female students (*B* = 2.444, *SE* = 1.216, *p* = 0.045, 95% CI: 0.060–4.827). No significant PIUS × sex interaction was observed for waist-to-hip ratio (*p* = 0.896), and the PIUS–WHR association did not reach significance within either sex stratum (male: *B* = 0.009, *p* = 0.474; female: *B* = 0.029, *p* = 0.057). These findings indicate that the association between problematic internet use and waist circumference, although detectable in both sexes, differs in magnitude by sex and should be interpreted with caution regarding its generalizability. Across the three primary linear regression models (Models 1–3), HC3 robust standard errors did not change the significance status of any predictor relative to the original ordinary-least-squares results; robust (sandwich-type) standard errors likewise left the significance pattern of Model 4 unchanged. Bootstrap 95% confidence intervals (1000 resamples) for the PFS total and PIUS total coefficients were closely consistent with the original estimates across all four models, and neither primary exposure changed significance status across the sensitivity analyses.

**Table 4:** Regression models predicting *BMI*, waist circumference, waist-to-hip ratio, and obesity status.

Variable	*B*	*β*	SE	*p*	OR	95% CI
Model 1. *Linear regression*—*BMI* (kg/m^2^)—*R*^2^ = 0.200, Adj. *R*^2^ = 0.174, *F* (8247) = 7.73, *p* < 0.001						
PFS total	1.321	0.204	0.398	0.001	—	[0.537, 2.105]
PIUS total	−0.388	−0.060	0.395	0.327	—	[−1.166, 0.390]
Sex (female)	−3.128	−0.317	0.603	<0.001	—	[−4.316, −1.940]
Exercise	−0.476	−0.049	0.595	0.425	—	[−1.648, 0.696]
Alcohol use	1.981	0.198	0.599	0.001	—	[0.801, 3.161]
Smoking	−0.805	−0.076	0.652	0.218	—	[−2.089, 0.479]
Age	0.247	0.140	0.127	0.052	—	[−0.003, 0.497]
Preclinical (ref. = clinical)	0.465	0.049	0.679	0.494	—	[−0.872, 1.802]
Model 2. *Linear regression*—*Waist circumference* (cm)—*R*^2^ = 0.402, Adj. *R*^2^ = 0.382, *F* (8247) = 20.71, *p* < 0.001						
PFS total	0.862	0.041	1.126	0.445	—	[−1.356, 3.080]
PIUS total	3.853	0.184	1.116	<0.001	—	[1.655, 6.051]
Sex (female)	−16.725	−0.519	1.705	<0.001	—	[−20.083, −13.367]
Exercise	−2.350	−0.074	1.681	0.163	—	[−5.661, 0.961]
Alcohol use	5.904	0.180	1.695	<0.001	—	[2.566, 9.242]
Smoking	−0.695	−0.020	1.843	0.706	—	[−4.325, 2.935]
Age	0.268	0.046	0.358	0.456	—	[−0.437, 0.973]
Preclinical (ref. = clinical)	2.531	0.081	1.919	0.188	—	[−1.249, 6.311]
Model 3. *Linear regression*—*Waist-to-hip ratio*—*R*^2^ = 0.357, Adj. *R*^2^ = 0.336, *F* (8247) = 17.12, *p* < 0.001						
PFS total	−0.018	−0.097	0.011	0.081	—	[−0.040, 0.004]
PIUS total	0.023	0.123	0.010	0.027	—	[0.003, 0.043]
Sex (female)	−0.149	−0.513	0.016	<0.001	—	[−0.181, −0.117]
Exercise	−0.005	−0.018	0.016	0.748	—	[−0.037, 0.027]
Alcohol use	0.052	0.176	0.016	0.001	—	[0.020, 0.084]
Smoking	0.033	0.106	0.017	0.057	—	[−0.000, 0.066]
Age	0.000	0.000	0.003	0.996	—	[−0.006, 0.006]
Preclinical (ref. = clinical)	−0.035	−0.123	0.018	0.055	—	[−0.070, 0.000]
Model 4. *Binary logistic regression*—*Overweight*/*obese* (*BMI* ≥ 25 = 1)—χ^2^(8) = 62.85, Nagelkerke *R*^2^ = 0.218, *p* < 0.001						
PFS total	0.581	—	—	0.014	1.788	[1.126, 2.839]
PIUS total	−0.194	—	—	0.391	0.824	[0.530, 1.282]
Sex (female)	−1.895	—	—	<0.001	0.150	[0.076, 0.296]
Exercise	−0.761	—	—	0.030	0.467	[0.236, 0.927]
Alcohol use	0.762	—	—	0.021	2.142	[1.119, 4.100]
Smoking	−0.062	—	—	0.859	0.940	[0.473, 1.866]
Age	0.138	—	—	0.047	1.148	[1.002, 1.316]
Preclinical (ref. = clinical)	0.432	—	—	0.251	1.541	[0.736, 3.225]

**Note.** Models 1–3 are linear regression; unstandardized coefficient (*B*), standardized coefficient (*β*), and standard error (SE) are reported. Model 4 is binary logistic regression; *B* and odds ratios (OR) with 95% confidence intervals (CI) are reported; *β* and SE are not applicable. PFS = Power of Food Scale total score; PIUS = Problematic Internet Use Scale total score; WHR = waist-to-hip ratio. Preclinical = Years 1–3; Clinical = Years 4–6. Bold = *p* < 0.05. For Models 1–3, the 95% CI corresponds to the unstandardized regression coefficient (*B*); for Model 4, the 95% CI corresponds to the odds ratio (OR).

## 4. Discussion

This cross-sectional study simultaneously evaluated hedonic hunger, problematic internet use (PIU), anthropometric measures, and lifestyle characteristics in medical students. Four principal findings emerged. First, hedonic hunger scores were positively associated with *BMI* and overweight/obesity status. Second, PIU demonstrated more consistent associations with waist circumference and waist-to-hip ratio than with *BMI*. Third, hedonic hunger and PIU scores were positively associated with each other. Fourth, regular exercise was associated with lower scores on both scales.

The mean PFS total score in our cohort was 3.25, which appears comparable to or slightly higher than values reported in previous studies conducted among Turkish university students [14,15,16]. Medical education has been associated with substantial academic workload, prolonged study periods, sleep disturbances, and psychological stressors that may influence health-related behaviors [17]. Although these variables were not directly measured in the present study, the relatively high PFS scores observed in our sample may warrant further investigation in future studies incorporating stress- and sleep-related assessments.

Consistent with the recent meta-analysis by Karamizadeh et al. [6], *BMI* was positively associated with PFS total score, particularly with the Food Available subscale. Similar associations between hedonic hunger, obesity-related measures, and food addiction have been reported in university-based populations [18,19,20]. In addition to correlation analyses, multivariable models in the present study demonstrated that higher PFS total scores were associated with higher *BMI* values and increased odds of overweight/obesity after adjustment for demographic and lifestyle characteristics. Hedonic hunger has been conceptualized as a behavioral construct encompassing homeostatic and reward-related aspects of food intake [4,21,22]. However, biological pathways potentially involved in hedonic eating behaviors were not evaluated in the present study, and mechanistic interpretations cannot be made from these findings.

An important aspect of the present findings is that different behavioral constructs were associated with different adiposity phenotypes. While hedonic hunger was associated with general adiposity (after adjustment for the measured covariates), reflected by *BMI* and overweight/obesity status, problematic internet use showed stronger associations with central adiposity indicators, particularly waist circumference and waist-to-hip ratio. Because waist circumference and waist-to-hip ratio are inherently influenced by sex and body size, this association should be interpreted with caution: sensitivity analyses revealed a significant PIUS × sex interaction for waist circumference, with a more pronounced association among male than female students, whereas no such interaction was observed for waist-to-hip ratio. These sex-specific patterns are considered exploratory and warrant replication in sex-balanced samples of adequate power. This finding differs from previous studies in university populations and from the meta-analysis by Aghasi et al. [9], which reported increased odds of overweight and obesity among individuals with greater internet use. Bao et al. similarly reported that internet addiction may coexist with obesity-related outcomes and psychological distress in medical students [10]. The reasons underlying the divergence between our central-adiposity-specific findings and these prior, largely *BMI*-focused reports cannot be determined from the present study; medical students represent a relatively homogeneous and health-literate population, and factors such as dietary habits, sleep quality, stress burden, academic internet use, and sedentary behaviors may have contributed to these observations [17,23], although these variables were not systematically assessed in the current study. Because central adiposity is more closely associated with cardiometabolic risk than *BMI* alone [11], the present findings suggest differential associations of hedonic hunger and problematic internet use with adiposity phenotypes in this population. Simultaneous assessment of these behavioral characteristics may provide complementary descriptive information when evaluating obesity-related health risks in young adults. As with all findings in this cross-sectional study, these observations should be interpreted as associations rather than as evidence of causal relationships. PFS and PIUS total scores were positively associated, with the strongest relationships observed between the Food Available and Food Present dimensions and PIUS total score. Similar associations between problematic internet use and eating-related psychopathology have been described in systematic reviews and meta-analyses conducted among student populations [24,25]. The present findings extend this literature by demonstrating associations between problematic internet use and hedonic dimensions of eating behavior in medical students. Nevertheless, given the cross-sectional design, it is not possible to determine the temporal sequence of these relationships or whether they are influenced by shared behavioral or environmental factors.

Regular exercise was associated with lower PFS total scores, lower PIUS total scores, and lower odds of overweight/obesity. Previous studies have reported favorable associations between physical activity and mental and behavioral health outcomes [26]. In our cohort, alcohol consumption was associated with higher adiposity measures in the multivariable models (Table 4) and, more selectively, with higher hedonic hunger scores in bivariate comparisons: this association was most robust for the Food Present subscale, whereas the PFS total and Food Available differences were only marginal or non-significant once Welch’s correction for unequal variances was applied (Table 3). Male students exhibited higher PIUS total, Social Benefit/Social Comfort, and Negative Consequences scores than female students. These observations are consistent with epidemiological studies reporting higher rates of problematic internet use among males [27]. However, the present findings should be interpreted as observational associations rather than evidence of causal relationships.

Several limitations should be acknowledged. First, the cross-sectional design precludes causal inference and does not permit assessment of the temporal sequence linking hedonic hunger, problematic internet use, and adiposity; longitudinal designs are required to clarify directionality. Second, participants were recruited from a single medical school, which may limit generalizability to other student populations, institutions, or cultural settings; in addition, the survey completion rate among eligible students was modest (25.3%), and because data on non-participants were not collected, the extent and direction of any participation bias could not be formally assessed. Furthermore, many bivariate correlations and subgroup comparisons were performed. Bonferroni correction was applied only to pairwise comparisons following a significant omnibus ANOVA, whereas correlations and other group comparisons were not adjusted for multiple testing. These secondary findings should therefore be interpreted cautiously. Third, and importantly, several potentially influential confounders were not systematically measured, including sleep quality and duration, dietary habits and eating patterns, psychological distress (e.g., depression, anxiety, and perceived stress), sedentary behavior and non-internet screen time, and socioeconomic status. Each of these factors is plausibly associated with both hedonic eating and problematic internet use, and their omission may have introduced residual confounding into the associations reported here; consequently, the reported effect sizes should be interpreted as unadjusted for these domains rather than as fully independent effects. Relatedly, physical activity was captured only as a binary self-reported item (regular exercise meeting a general moderate-/vigorous-intensity threshold: yes/no) rather than with a validated instrument such as the International Physical Activity Questionnaire (Short Form), precluding assessment of activity frequency, duration, or dose–response relationships. Fourth, all anthropometric measurements were performed by a single trained internal medicine physician; the first 10 students were measured jointly by two observers to confirm measurement consistency before proceeding, but a formal inter-rater or intra-rater reliability coefficient (e.g., intraclass correlation) was not calculated for the full sample, and day-to-day measurement variability could not be assessed. Fifth, problematic internet use was assessed using a self-report instrument that does not distinguish academic internet use from recreational internet use, which may have attenuated or obscured associations specific to recreational use. Finally, although Bonferroni-adjusted post hoc analyses, multivariable regression models, and reliability and multicollinearity diagnostics were applied to strengthen the rigor of the analysis, replication in larger, multicenter, and longitudinal cohorts incorporating the unmeasured variables noted above is warranted before these findings are generalized.

## 5. Conclusions

This cross-sectional study examined hedonic hunger, problematic internet use, and multiple adiposity indices in 256 medical students. Multivariable regression analyses demonstrated differential associations: hedonic hunger, measured by the Power of Food Scale, was associated with higher *BMI* and greater odds of overweight/obesity after adjustment for the measured demographic and lifestyle covariates, whereas PIU scores were more consistently associated with waist circumference and waist-to-hip ratio but not with *BMI*. These findings suggest that hedonic hunger and PIU show differential cross-sectional associations with general (*BMI*) versus central (waist circumference and waist-to-hip ratio) measures of adiposity in this population. Hedonic hunger and PIU scores were positively correlated, and regular exercise was associated with lower scores on both scales. These findings should be interpreted as exploratory cross-sectional associations. Future longitudinal studies are required to clarify the temporal relationships among these variables and to determine whether the observed associations persist over time.

## Data Availability

The data supporting the findings of this study are openly available in Zenodo at https://doi.org/10.5281/zenodo.21218265 (accessed on 26 June 2026).

## Supplementary Materials

The following supporting information can be downloaded at: https://www.mdpi.com/article/10.3390/healthcare14162483/s1, Table S1: PFS and PIUS scores according to academic year; Table S2: Comparison of PFS, PIUS, *BMI*, and lifestyle variables between preclinical and clinical students; Table S3: Pearson’s correlations of age and internet use duration with PFS and PIUS scores; Table S4: Comparison of Power of Food Scale and Problematic Internet Use Scale scores across *BMI* categories.
