# Supplementary material for: Hedonic Hunger and Problematic Internet Use Show Distinct Associations with General and Central Adiposity in Medical Students
Source: Healthcare (Basel). 2026 Aug 11;14(16):2483. doi: 10.3390/healthcare14162483 (PMC13512366; doi:10.3390/healthcare14162483)
Supplement: Supplementary file 1 [file healthcare-14-02483-s001.zip › healthcare-4433409-supplementary.pdf]

Supplementary Data

Supplementary Table S1. PFS and PIUS scores according to academic year.

	Year 1	Year 2	Year 3	Year 4	Year 5	Year 6	F	*p*	Post Hoc
**Variable**	**(n = 31)**	**(n = 50)**	**(n = 62)**	**(n = 34)**	**(n = 17)**	**(n = 62)**			
PFS total	3.29	3.25	3.42	3.21	2.84	3.21	1.82 (η^2p = 0.035)	0.109	ns
PFS-FA	3.00	2.86	3.07	2.83	2.50	2.75	1.55 (η^2p = 0.030)	0.176	ns
PFS-FP	3.35	3.32	3.54	3.27	3.09	3.33	0.96 (η^2p = 0.019)	0.442	ns
PFS-FT	**3.51**	**3.57**	**3.65**	**3.51**	**2.94**	**3.54**	**2.29** (η^2p = 0.044)	**0.047**	Year 2, Year 3 > Year 5 [a g]
PIUS total	2.49	2.55	2.71	2.74	2.16	2.65	1.97 (η^2p = 0.038)	0.084	ns
PIUS-EU	**3.33**	**3.44**	**3.46**	**3.56**	**2.78**	**3.53**	**2.26** (η^2p = 0.043)	**0.049**	ns [† h]
PIUS-SB	2.18	2.09	2.35	2.29	1.93	2.15	1.14 (η^2p = 0.022)	0.340	ns
PIUS-NC	1.98	2.12	2.33	2.37	1.76	2.26	1.81 (η^2p = 0.035)	0.112	ns

Note. Values are mean scores. One-way ANOVA with Bonferroni post hoc (15 comparisons). PFS = Power of Food Scale; PIUS = Problematic Internet Use Scale; FA = Food Available; FP = Food Present; FT = Food Tasted; EU = Excessive Use; SB = Social Benefit/Social Comfort; NC = Negative Consequences. Bold = significant omnibus F ($p < 0.05$). [†] Significant F but no individual pair survived Bonferroni correction. [a] Bonferroni-corrected $p < 0.05$. ns = not significant. [g] Robust to sensitivity analysis (Welch's ANOVA p = 0.014; Kruskal–Wallis $p = 0.021$). [h] Omnibus F did not remain significant under Welch's ANOVA ($p = 0.172$) or Kruskal–Wallis test ($p = 0.130$), and the small Year 5 subgroup (n = 17); this finding should not be treated as confirmatory. η^2p = partial eta-squared.

Supplementary Table S2. Comparison of PFS, PIUS, BMI, and lifestyle variables between preclinical and clinical students.

Variable	Preclinical (n = 143)	Clinical (n = 113)	*p*
PFS total	3.33 ± 0.72	3.15 ± 0.75	0.051 (d = 0.25)
PFS-FA	**2.98 ± 0.90**	**2.74 ± 0.91**	**0.031** (d = 0.27)
PFS-FP	3.42 ± 0.87	3.28 ± 0.89	0.195 (d = 0.16)
PFS-FT	3.59 ± 0.73	3.44 ± 0.84	0.127 (d = 0.19)
PIUS total	2.61 ± 0.72	2.60 ± 0.77	0.939 (d = 0.01)
PIUS-EU	3.42 ± 0.84	3.42 ± 0.93	0.996 (d = −0.00)
PIUS-SB	2.22 ± 0.80	2.16 ± 0.81	0.563 (d = 0.07)
PIUS-NC	2.18 ± 0.86	2.22 ± 0.95	0.749 (d = −0.04)
**BMI (kg/m²)**	**23.07 ± 5.17**	**24.55 ± 4.06**	**0.013** (d = −0.31)
**Above normal BMI, n (%)**	**34 (23.8%)**	**48 (42.5%)**	**0.002**
**Smoking, n (%)**	**31 (21.7%)**	**41 (36.3%)**	**0.015**
Alcohol use, n (%)	43 (30.1%)	44 (38.9%)	0.176
Regular exercise, n (%)	50 (35.0%)	52 (46.0%)	0.096

Note. Values are mean ± SD unless otherwise stated. Independent-samples *t*-test for continuous variables; Chi-square test for categorical variables. Preclinical = Years 1–3; Clinical = Years 4–6. Above normal BMI = BMI ≥ 25 kg/m². PFS = Power of Food Scale; PIUS = Problematic Internet Use Scale; FA = Food Available; FP = Food Present; FT = Food Tasted; EU = Excessive Use; SB = Social Benefit/Social Comfort; NC = Negative Consequences. Bold = $p < 0.05$. d = Cohen's d, reported for continuous outcomes (rows 1–9).

Supplementary Table S3. Pearson's correlations of age and internet use duration with PFS and PIUS scores.

	Age		Internet Use Duration (Years)	
**Variable**	**r**	***p***	**r**	***p***
**PFS total**	**−0.172**	**0.006**	0.035	0.580
PFS-FA	**−0.176**	**0.005**	−0.030	0.630
PFS-FP	**−0.131**	**0.036** [i]	0.094	0.133
PFS-FT	**−0.131**	**0.037**	0.026	0.674
**PIUS total**	−0.037	0.556	**0.181**	**0.004**
PIUS-EU	−0.056	0.370	0.079	0.209
PIUS-SB	−0.035	0.574	**0.178**	**0.004**
PIUS-NC	−0.005	0.936	**0.211**	**<0.001**

Note. Pearson's correlation coefficients (r) are shown. Internet use duration = years of active internet use (M = 11.3, range 3–28). PFS = Power of Food Scale; PIUS = Problematic Internet Use Scale; FA = Food Available; FP = Food Present; FT = Food Tasted; EU = Excessive Use; SB = Social Benefit/Social Comfort; NC = Negative Consequences. Bold = $p < 0.05$. [i] Not confirmed with Spearman's rank-order correlation (age–PFS-FP: $\varrho = -0.104$, $p = 0.098$); this correlation should be interpreted with caution.

Supplementary Table S4. Comparison of Power of Food Scale and Problematic Internet Use Scale scores across BMI categories.

Variable	Normal/Underweight	Overweight	Obese	F	*p*	Post Hoc
BMI (kg/m²)	21.26 (2.27)	27.14 (1.43)	33.84 (3.72)	—	—	—
**Waist circumference (cm)**	**72.60 (10.82)**	**90.18 (11.50)**	**98.00 (20.66)**	**63.79** [j] (η^2p = 0.381)	**<0.001**	1 < 2 [b]; 1 < 3 [b]
**Hip circumference (cm)**	**90.18 (11.42)**	**99.79 (11.09)**	**108.35 (14.11)**	**35.96** (η^2p = 0.221)	**<0.001**	1 < 2 < 3 [a]
**Waist-to-hip ratio**	**0.814 (0.132)**	**0.907 (0.092)**	**0.918 (0.200)**	**18.30** [j] (η^2p = 0.104)	**<0.001**	1 < 2 [b]; 1 < 3 [b]
**PFS total**	**3.175 (0.709)**	**3.300 (0.754)**	**3.671 (0.741)**	**5.49** (η^2p = 0.042)	**0.005**	1 < 3 [a]; 2 < 3 [b]
Food available (FA)	**2.738 (0.877)**	**3.033 (0.920)**	**3.442 (0.835)**	**8.38** (η^2p = 0.062)	**<0.001**	1 < 2 [b]; 1 < 3 [a]
Food present (FP)	**3.257 (0.867)**	**3.397 (0.817)**	**3.962 (0.916)**	**7.64** (η^2p = 0.057)	**<0.001**	1 < 3 [a]; 2 < 3 [b]
Food tasted (FT)	3.530 (0.746)	3.471 (0.948)	3.608 (0.661)	0.28 (η^2p = 0.002)	0.759	ns
PIUS total	2.563 (0.674)	2.585 (0.799)	2.933 (0.976)	1.72 [j] (η^2p = 0.022)	0.188 [j]	ns
Excessive use	3.454 (0.789)	3.333 (1.091)	3.423 (1.006)	0.39 (η^2p = 0.003)	0.675	ns
Perceived social benefit	**2.113 (0.775)**	**2.245 (0.742)**	**2.631 (0.952)**	**5.04** (η^2p = 0.038)	**0.007**	1 < 3 [a]
Negative Consequences (PIUS-NC)	**2.120 (0.826)**	**2.178 (0.887)**	**2.744 (1.227)**	**3.12** [j] (η^2p = 0.043)	**0.052** [j]	ns (Welch) [j]

Note. Values are presented as M (SD). PFS = Power of Food Scale; PIUS = Problematic Internet Use Scale. One-way ANOVA with Bonferroni-corrected post hoc comparisons. Group 1 = Normal/Underweight (BMI < 25 kg/m²); Group 2 = Overweight (BMI 25–29.9 kg/m²); Group 3 = Obese (BMI ≥ 30 kg/m²). Bold rows indicate significant omnibus F (p < 0.05). [j] Levene's test indicated unequal variances for this outcome; Welch's ANOVA (F, p) is reported here in place of the original classical ANOVA, with Games–Howell post hoc comparisons in place of Bonferroni where applicable. For waist circumference, the overweight-vs-obese pairwise difference was not significant under Games–Howell correction ($p = 0.184$). For the Negative Consequences (PIUS-NC) score, the omnibus difference did not remain significant under Welch's ANOVA and is therefore not interpreted as a robust finding. η^2p = partial eta-squared. [a] $p < 0.01$ (Bonferroni-corrected). [b] $p < 0.05$ (Bonferroni-corrected). ns = not significant.
